# No difference in sudden‐onset injury risk between artificial turf and natural grass for Finnish female elite‐level footballers: A five‐season study

**DOI:** 10.1002/ksa.70018

**Published:** 2025-08-31

**Authors:** Ville Immonen, Iida Mustakoski, Ilari Kuitunen, Tommi Vasankari, Mari Leppänen

**Affiliations:** ^1^ Institute of Clinical Medicine University of Eastern Finland Kuopio Finland; ^2^ Tampere Research Center of Sports Medicine UKK Institute Tampere Finland; ^3^ University of Jyväskylä Jyväskylä Finland; ^4^ UKK Institute Tampere Finland; ^5^ Faculty of Medicine and Health Technology Tampere University Tampere Finland; ^6^ Tampere University Hospital Tampere Finland

**Keywords:** artificial turf, female, football, injury, playing surface, sports medicine

## Abstract

**Purpose:**

Evidence on injury incidence on artificial turf for female footballers is conflicting. Some studies have found no difference in injury rates, while others have suggested increased knee injury risk. The aim of this study was to compare match injury incidences between artificial turf and natural grass in the Finnish female premier division of football.

**Methods:**

All teams in the Finnish female premier division of football were invited to participate in a five‐season prospective cohort study, and eight to ten teams took part depending on the season. Injuries were reported by players in weekly questionnaires and categorised by anatomical region, recurrence, contact, severity, and playing position. Individual match exposure was tabled, and incidences per 1000 h of match exposure and incidence rate ratios (IRRs) with 95% confidence intervals (CIs) were calculated for both surface types.

**Results:**

A total of 517 league matches (401 on artificial turf and 116 on natural grass) were played during the five‐season follow‐up. In that time, 237 sudden‐onset injuries (184 on artificial turf and 53 on natural grass) were reported. The overall injury incidence rate was 19.6/1000 match hours on artificial turf and 19.3/1000 match hours on natural grass (IRR 1.0, 95% CI 0.7–1.4). No statistical difference was observed for risk in knee injuries or other subcategories.

**Conclusions:**

This study found no evidence of a difference in match injury risk between artificial turf and natural grass for elite level female footballers. Research with modern non‐filler surfaces will be needed as pitches containing microplastic pollution are banned in the European Union.

**Level of Evidence:**

Level II.

**Abbreviations**
ACL: anterior cruciate ligament
CI: confidence interval
FIFA: The International Football Federation
IRR: incidence rate ratio
SD: standard deviation

## INTRODUCTION

Using artificial turf instead of natural grass as a playing surface in football offers multiple practical advantages, such as higher usage rates, weather resistance, no requirement for sunlight, and the possibility of multi‐use pitches without damaging the surface [5]. Initial trials in the 1980s with turf pitches in professional football were unsuccessful because the quality of the game was considered to decrease significantly. Not until 2004 did the International Football Association Board approved artificial turf surfaces for international competitions. The International Football Federation (FIFA) has since created a quality programme to identify turf pitches, and it demands that artificial turfs use a third‐generation system where the infill contains both sand and rubber material [5].

Questionnaires have shown that professional footballers generally have negative perceptions of artificial turf and consider it a major injury risk factor [2, 9, 21]. However, studies have not demonstrated increased overall injury risk on artificial turf. A recent meta‐analysis actually showed a decrease in overall injury risk compared to natural grass [14]. Some investigations have suggested an increased risk for injury in female footballers on artificial turf [17, 20, 22, 29], and female players used injury concerns as an argument in a discrimination case against FIFA concerning the 2015 World Cup in Canada where artificial surface was used. The evidence for increased injury risk is conflicting, however, as multiple studies have also shown no difference [4, 7, 8, 10] or a decrease [11, 14, 16, 19] in injury risk for female footballers on artificial turf.

Injury risk factors in football have generally been studied with male footballers, especially with professionals. Most studies investigating the effect of playing surface on injury risk for female footballers have been conducted in the United States or a Nordic country. The few studies focusing on Northern Europe considered mainly amateur and youth players [10, 23, 24]. To our knowledge, only one European study has investigated injury risk on artificial turf for female elite level footballers [4]. That study found no difference in acute injuries between the surfaces. In Finland, injury risk per surface was recently studied with elite level male footballers [12] but not with females.

The aim of this study was to compare the injury risk between natural grass and artificial turf in the female premier football division of Finland during seasons 2020–2024.

## METHODS

### Study design

This study is part of five‐season prospective cohort study among female football players in Finland. The current sub‐study investigated the effect of playing surface on sudden‐onset injury risk.

### Study population

All ten teams of the Finnish female premier division of football were invited to participate in the follow‐up in each of the five seasons during 2020–2024. Eight teams agreed to participate in each season during 2020–2023, and all ten teams participated in the 2024 season. Teams that did not participate in individual seasons had their own injury follow‐up system at the time. Depending on the season, two or three participating teams played their home matches on natural grass for the whole season or part of it. The other five to seven participating teams played all their home matches on artificial turf. Teams whose home surface changed during the season played primarily on natural grass but were forced to start and/or end their season on artificial turf due to weather conditions. Season characteristics are presented in Table 1.

**Table 1:** Season characteristics.

	2020	2021	2022	2023	2024
Follow‐up period	May–Nov	Jan–Oct	Jan–Oct	Mar–Oct	Jun–Oct
Number of follow‐up weeks	24	40	38	29	20
Weekly questionnaire response rate (mean)	95%	85%	83%	87%	75%
Number of participating teams	8	8	8	8	10
Number of players in the follow‐up	158	171	173	154	205
Surface in the home field (number of teams)					
Artificial turf	5	5	5	6	7
Natural grass	2	0	0	0	0
Alternate surface^a^	1	3	3	2	3
Number of league matches during the follow‐up period (hours of reported exposure)					
Artificial turf	56 (1166)	90 (2056)	91 (2253)	102 (2131)	62 (1762)
Natural grass	34 (637)	27 (642)	20 (570)	15 (361)	20 (560)

^a^ Team played primarily on natural grass but started and/or ended their season on artificial turf.

All players with official team membership and aged ≥ 15 years were able to participate. During the 2023 season, only players aged ≥ 18 years were invited to participate in the study. Written informed consent was obtained from each player prior to participation. Participation was voluntary, and participants had the right to withdraw or decline consent at any time with no disadvantage. The parents or legal guardians of underaged participants were informed about the study.

### Data collection

Injury data were collected between May 2020 and October 2024 and included five separate follow‐up periods, one for each season (Table 1). Normally, the female premier division season in Finland plays from late March or early April to late October. The start of the 2020 season was postponed due to the COVID‐19 pandemic and therefore ended later than usual. The follow‐up period for the 2024 season started exceptionally in June because the monitoring application was changed during the season.

At the beginning of each season, participants completed a questionnaire collecting basic demographic information on an online platform (Webropol, Finland). During the season, injury data were collected using a mobile application (AthleteMonitoring, Canada/XPS Network, Iceland) where players completed a weekly health questionnaire. Average weekly response rates varied from 75% to 94% depending on the season (Table 1). Missing information was completed using phone interviews, and injury details were crosschecked and completed with final survey answers and by asking team physiotherapists.

An injury was defined per the Walden consensus as ‘tissue damage or other derangement of normal physical function, resulting from rapid or repetitive transfer of kinetic energy’ [28]. All physical complaints resulting from participation in football, irrespective of the need for medical attention or time loss, were registered. Injuries were further categorised into sudden‐onset and gradual‐onset injuries. Only sudden‐onset injuries occurring in league matches were included in the current analysis. A sudden‐onset injury was defined as an injury resulting from a single specific event.

Injuries were categorised according to body region following the Bahr consensus [3]. Injury severity was based on the number of days that the player was unavailable for full training and matches [3]. The following severity categories were used in reporting: 0, 1–3, 4–7, 8–28, 29–90, 91–180 and >180 days [28]. Recurrent injury was defined as injury of the same type at the same location as the original injury, occurring after the player's return to full participation between the injuries. Injuries were also categorised per contact as non‐contact injuries (no disruption in player's movement pattern), indirect contact injuries (contact to a body part other than the one in which the injury occurred), and direct contact injuries (contact to the injured body part) [3, 28]. If the contact information was missing but the injury mechanism and location were precisely described, the contact information was completed. Injury types were also registered but are not included in this report since they were not confirmed by a medical professional. Anterior cruciate ligament (ACL) injuries were diagnosed by a medical doctor and are therefore included as an individual diagnosis.

All reported sudden‐onset league match injuries were initially included in the study. If the playing surface information was missing but the injury date was known, data were completed based on match schedule and stadium surface information. If both the injury date and the playing surface information were missing, the injury was excluded from the study. Pitch standards in the Finnish female premier division of football are determined by the Football Association of Finland, who require approval of the government licensing office for all artificial surfaces used in the league [25]. Many of the pitches used in the female premier division are also used in the male premier division, which demands FIFA Quality Concept of Football Turf or FIFA Quality Pro Standard requirements to be met [26]. In general, all artificial turfs used in competitive football in Finland are at least third‐generation turf systems.

Individual match exposure and players' typical positions were collected based on player information at Flashscore.com and the official league website. Player position categories in this study were goalkeeper, defender (including center‐backs and full‐backs), midfielder, and striker (including wingers and forwards). The surface for each league match was checked in the match schedule and also from a television broadcast in uncertain cases. Individual match exposures were tabled and summarised for both surfaces to calculate overall match exposure.

### Statistical analysis

Team and season characteristics are presented as absolute values or percentages and player characteristics as means and standard deviations (SDs). Categorised variables are presented as absolute values and proportions. Injury incidence was calculated per 1000 h of match exposure. We used incidence rate ratios (IRRs) to compare artificial turf to natural grass. The Poisson exact method was used to calculate 95% confidence intervals (CIs). We used stratified analyses as player attributable factors cannot be considered confounders (Supporting Information: Figure 1).

## RESULTS

Depending on the season, 154–205 players participated in the follow‐up (Table 1). The mean age of all players was 21.2 years (median 20, range 15–34), and they had played 3.6 seasons (median 3, range 1–18) in the league. The mean height was 167.8 centimeters (SD 5.8), and the mean weight was 64.0 kilograms (SD 7.9). Player characteristics per season are presented in Table 2.

**Table 2:** Player characteristics (mean (SD)).

	2020	2021	2022	2023	2024
Age	21.2 (3.5)	21.3 (3.5)	21.2 (3.6)	21.2 (3.1)	21.0 (3.4)
Height (cm)	167.5 (6.3)	168.2 (6.2)	167.4 (5.7)	167.5 (5.1)	168.1 (5.3)
Weight (kg)	64.0 (10.7)	63.8 (8.4)	64.0 (6.7)	64.1 (6.0)	64.1 (6.3)
How many seasons in the premier division (including the following season)	4.0 (3.2)	3.7 (3.1)	3.3 (2.8)	3.6 (2.6)	3.3 (2.6)

During the five‐season follow‐up, 401 league matches were played on artificial turf and 116 matches on natural grass (Table 1). In these matches, study participants recorded 9368 hours of match exposure on artificial turf and 2770 h on natural grass. A total of 244 league match injuries were reported, of which playing surface was originally known in 190 cases. The playing surface information was added for 47 injuries, and 7 injuries were excluded for missing both the injury date and the playing surface information. Altogether, 237 injuries were included in the study, of which 184 occurred on artificial turf and 53 on natural grass.

**Figure 1:**
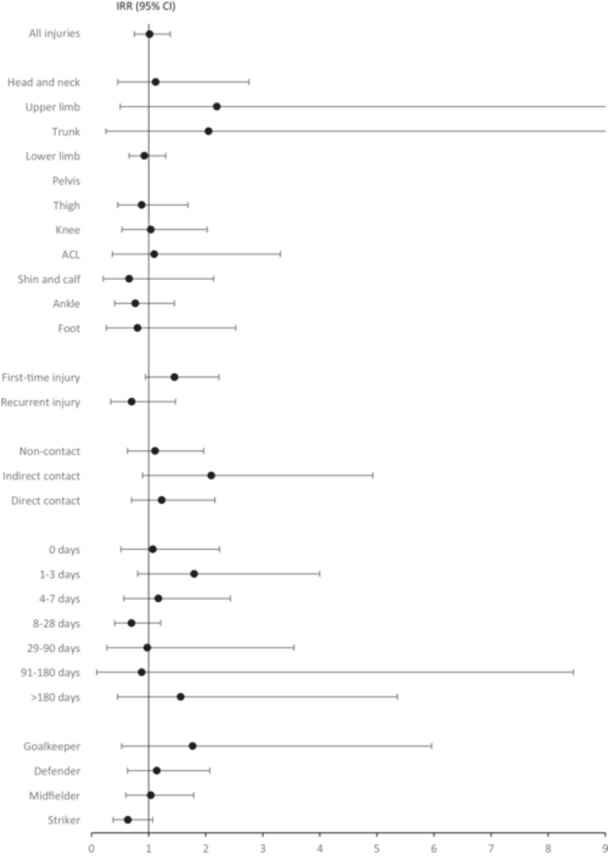
Incidence rate ratios (IRRs) representing the injury incidence per 1000 h of match exposure on artificial turf compared to natural grass. IRR lower than 1.0 would indicate lower risk for injuries on artificial turf and IRR higher than 1.0 would indicate higher risk for injuries on artificial turf compared to natural grass.

The overall injury incidence rate was 19.6/1000 h of match exposure on artificial turf and 19.3/1000 match hours on natural grass (IRR 1.0, 95% CI 0.7–1.4) (Table 3, Figure 1).

**Table 3:** Number of injuries (*n*) and injury incidence per 1000 h of match exposure on artificial turf and natural grass.

	Artificial turf		Natural grass		
	*n*	Incidence (95% CI)	*n*	Incidence (95% CI)	IRR (95% CI)
All injuries	184	19.6 (16.9–22.6)	53	19.3 (14.6–25.0)	1.0 (0.7–1.4)
Anatomical region					
Head and neck	23	2.4 (1.6–3.6)	6	2.2 (0.9–4.5)	1.1 (0.5–2.8)
Shoulder, arm and hand	15	1.6 (0.9–2.6)	2	0.7 (0.1–2.3)	2.2 (0.5–9.6)
Chest, spine and abdomen	7	0.7 (0.3–1.5)	1	0.4 (0.0–1.7)	2.0 (0.3–16.7)
Lower limb	139	14.8 (12.5–17.4)	44	16.0 (11.8–21.3)	0.9 (0.7–1.3)
Hip and groin	10	1.1 (0.5–1.9)	0	‐	‐
Thigh	36	3.8 (2.7–5.2)	12	4.4 (2.4–7.4)	0.9 (0.5–1.7)
Knee	39	4.2 (3.0–5.6)	11	4.0 (2.1–6.9)	1.0 (0.5–2.0)
*ACL*	*15*	*1.6 (0.9–2.6)*	*4*	*1.5 (0.5–3.5)*	*1.1 (0.4–3.3)*
Lower leg	9	1.0 (0.5–1.7)	4	1.5 (0.5–3.5)	0.7 (0.2–2.1)
Ankle	34	3.6 (2.6–5.0)	13	4.7 (2.6–7.9)	0.8 (0.4–1.5)
Foot	11	1.2 (0.6–2.0)	4	1.5 (0.5–3.5)	0.8 (0.3–2.5)
Injury recurrence					
First‐time injury	124	13.2 (11.0–15.7)	25	9.1 (6.0–13.2)	1.5 (0.9–2.2)
Recurrent injury	24	2.6 (1.7–3.7)	10	3.6 (1.9–6.5)	0.7 (0.3–1.5)
Missing information	36		18		
Contact					
Non‐contact	57	6.1 (4.6–7.8)	15	5.5 (3.2–8.8)	1.1 (0.6–2.0)
Indirect contact	43	4.6 (3.4–6.1)	6	2.2 (0.9–4.5)	2.1 (0.9–4.9)
Direct contact	63	6.7 (5.2–8.5)	15	5.5 (3.2–8.8)	1.2 (0.7–2.2)
Missing information	21		17		
Severity					
0 days	33	3.5 (2.5–4.9)	9	3.3 (1.6–6.0)	1.1 (0.5–2.2)
1–3 days	43	4.6 (3.4–6.1)	7	2.5 (1.1–5.0)	1.8 (0.8–4.0)
4–7 days	36	3.8 (2.7–5.2)	9	3.3 (1.6–6.0)	1.2 (0.6–2.4)
8–28 days	43	4.6 (3.4–6.1)	18	6.5 (4.0–10.1)	0.7 (0.4–1.2)
29–90 days	10	1.1 (0.5–1.9)	3	1.1 (0.3–2.9)	1.0 (0.3–3.5)
91–180 days	3	0.3 (0.1–0.9)	1	0.4 (0.0–1.7)	0.9 (0.1–8.4)
>180 days	16	1.7 (1.0–2.7)	3	1.1 (0.3–2.9)	1.6 (0.5–5.4)
Missing information	0		3		
Position					
Goalkeeper	20	23.4 (14.7–35.4)	3	13.2 (3.7–35.2)	1.8 (0.5–6.0)
Defender	48	16.1 (12.0–21.2)	14	14.1 (8.1–23.1)	1.1 (0.6–2.1)
Midfielder	67	18.0 (16.3–26.3)	16	17.3 (10.3–27.5)	1.0 (0.6–1.8)
Striker	49	21.0 (15.7–27.5)	20	32.9 (20.7–49.8)	0.6 (0.4–1.1)

Abbreviation: CI, confidence interval.

The incidence rate for all lower limb injuries was 14.8/1000 match hours on artificial turf and 16.0/1000 match hours on natural grass (IRR 0.9, 95% CI 0.7–1.3). The majority of injuries occurred in the thigh, knee, or ankle area. The incidence rate of ACL injuries was 1.6/1000 match hours on artificial turf and 1.5/1000 match hours on natural grass (IRR 1.1, 95% CI 0.4–3.3). There was no difference in injury risk between the surfaces in any anatomical region (Table 3, Figure 1).

The incidence rate for recurrent injuries was 2.6/1000 match hours on artificial turf and 3.6/1000 match hours on natural grass (IRR 0.7, 95% CI 0.3–1.5). The incidence rate for first‐time injuries was 13.2/1000 match hours on artificial turf and 9.1/1000 match hours on natural grass (IRR 1.5, 95% CI 0.9–2.2). Injury recurrence information was missing from 23% of total injuries (36 on artificial turf, 18 on natural grass) (Table 3, Figure 1).

The incidence rate for indirect contact injuries was 4.6/1000 match hours on artificial turf and 2.2/1000 match hours on natural grass (IRR 2.1, 95% CI 0.9–4.9). There was no significant difference in non‐contact or direct contact injuries. Injury contact information was completed for 10 injuries based on the injury mechanism and location. Injury contact information was missing from 16% of total injuries (21 on artificial turf, 17 on natural grass) (Table 3, Figure 1).

The most common category of injury severity was 8–28 day absence (4.6/1000 match hours on artificial turf versus 6.5/1000 match hours on natural grass, IRR 0.7, 95% CI 0.4–1.2). There was no significant difference in injury severity between the surfaces. Injury severity information was missing from 1.3% of total injuries (none on artificial turf, three on natural grass) (Table 3, Figure 1).

In positional comparison, goalkeepers had the most injuries on artificial turf (23.4/1000 match hours), and strikers had the most injuries on natural grass (32.9/1000 match hours). There was no statistical difference in injury risk according to playing position (Table 3, Figure 1).

## CONCLUSIONS

No meaningful difference in overall injury risk was observed between artificial turf and natural grass for female premier division footballers. This result is in line with previous studies made with female elite level players that have found overall injury risk to be either similar [4, 7] or lower [16] on artificial turf.

Anatomically, there were no significant differences in injury risk in any category. A meta‐analysis and an epidemiological study found the risk of ACL injuries to be higher on artificial turf in female football [17, 29]. On the other hand, another recent meta‐analysis and two original studies reported no difference in ACL or knee injuries for female players according to surface type [10, 11, 14], and the topic has remained uncertain. In this study, there was no difference in overall knee injury risk or ACL injury risk. Interest in factors affecting knee injuries is currently high in sports traumatology, and recent studies have suggested significant factors other than athletes' physical attributes that associate with risk of knee injury, such as psychological features [15, 18].

In this population, first‐time injuries were more frequent on artificial turf, but the result was not statistically significant. Also, information about injury recurrence was missing from multiple injury reports on both surfaces. The same applies to indirect contact injuries, which were more frequent on artificial turf in this study, but the result did not reach statistical significance. No difference in injury risk between the surfaces was found in injury severity. Only a few studies have investigated these injury subcategories for female footballers. They found no difference in injury recurrence [16, 24] or contact mechanism [7, 8, 24] with female players in training or match play according to surface. Evidence of injury severity has been more conflicting, as a single article observed the incidence of serious injuries to be higher on artificial turf with youth female players [24], while most studies have found either no difference in injury severity [4, 7, 8] or lower incidence of substantial injuries on artificial turf [16].

No statistical difference in injury risk per playing position was found between the surfaces in this study. Strikers had fewer injuries on artificial turf, but this finding was not statistically significant. To our knowledge, only one previous study has investigated the effect of playing position on injury risk according to surface among female footballers. That study found injuries to happen less frequently for offensive players on artificial turf although there was no significant difference when offensive players were compared to defensive players [16]. In contrast, a recent study investigating Finnish male elite level footballers concluded that injury risk on artificial turf might increase for strikers [12]. The evidence on this topic remains unclear.

The relatively long follow‐up period is a significant strength of this study. To our knowledge, this is only the second original study to investigate the correlation between playing surface and injuries with European female footballers playing at the premier division level, although it is notable that most Finnish female league players are not professional footballers. In the previous study, Ekstrand et al. followed mainly male teams, and only five female teams were involved [4]. Since that study, artificial turf materials have improved significantly and FIFA has updated its requirements for football pitches several times [6].

Several external factors, such as weather conditions, may have influenced the results of this study. A recent study with youth footballers conducted in Denmark indicated that traumatic injuries are increased in drier field conditions [13]. According to Finnish Meteorological Institute, the usual annual precipitation in Finland varies between 500 and 700 millimetres in areas where premier division football is played. This is for example slightly below the average of whole Europe or the United States [27]. Apart from field dryness, very little is known about the effect of weather on injury risk in football. The temperature in Finland varies considerably depending on the year especially in spring, which means that football pitches may sometimes be exposed to ice and snow at the start of the season. This naturally affects the condition of natural grass pitches but may also have an effect on the attributes of artificial surfaces.

Injury types were not included in this study, which can be seen as a limitation. The number of injuries in most subgroups was relatively low, which adds imprecision to the results. Also, teams' training surfaces and individual training exposure during the follow‐up were not known, and therefore, training injuries and exposure could not be included in the study. In addition, the follow‐up period in 2024 did not cover the whole season because of the change in the monitoring application. In the 2024 season, the response rate to the weekly questionnaire was slightly lower compared to previous seasons, also possibly due to the change in the application used. However, the season's response rate of 75% is still somewhat similar to that of a study among Norwegian players using the same methodology [1].

This study is in line with previous evidence that artificial turf does not increase the overall injury risk for elite level female footballers. Based on this study, claims that artificial turf increases the prevalence of knee injuries among female players should also be questioned. More research on the topic is needed especially with female elite level players. In the near future, most artificial turfs in Europe will face mandatory material change, as the European Union is banning turf fillers categorised as microplastic pollution, and for example Finland and Belgium have started to test artificial non‐filler surfaces. During this transition, it is important to obtain information about the effect of the new surface for footballers.

## AUTHOR CONTRIBUTIONS

All authors have contributed to the study.

## CONFLICT OF INTEREST STATEMENT

The authors declare no conflicts of interest.

## ETHICS STATEMENT

Approval for the study design and methods was obtained from Ethics Committee of Pirkanmaa Hospital District (Tampere, Finland) (code R19135). Written informed consent was obtained from all individual participants included in the study.

## Supporting information

Supporting Figure 1

## Data Availability

The data that support the findings of this study are available on reasonable request from the corresponding author. The data are not publicly available due to information that could compromise the privacy of research participants.
